# Idiopathic Cutis Verticis Gyrata in a Female

**DOI:** 10.7759/cureus.2105

**Published:** 2018-01-23

**Authors:** Mohamed amine Ennouhi, Alae Guerrouani, Abdennacer Moussaoui

**Affiliations:** 1 Plastic and Reconstructive Surgery Unit, Moulay Ismail Military Hospital Meknes; 2 Departement of Oral and Maxillofacial Surgery, Dalhousie University

**Keywords:** cutis verticis gyrata, scalp, surgery

## Abstract

Cutis verticis gyrata (CVG) is a benign proliferation and hypertrophy involving the scalp which exhibits furrows resembling the cerebral cortex convolutions. The diagnosis of CVG is based on clinical findings. Complementary investigations are recommended to rule out local or systemic underlying disorders.

Idiopathic or essential primary CVG refers to cases without obvious origin and with no other associated abnormalities. These forms affect mainly men. Herein, the authors present a new observation of an idiopathic CVG in a female, which seems to be extremely rare.

## Introduction

First described by Alibert in 1837 [1], cutis verticis gyrata (CVG), also called "bulldog scalp or corrugated scalp'' [2, 3], is a rare, benign and progressively evolving scalp deformity characterized by an excessive proliferation and hypertrophy of the skin and the subcutaneous tissue. The affected area exhibits folds and furrows resembling the convolutions of the cerebral cortex. CVG may be primary or secondary. Relatively rare, primary forms occur in men while secondary CVGs affect both sexes. To our knowledge, the present observation is about the second case of idiopathic CVG in females.

## Case presentation

A 36-year-old female with no significant medical history was referred to our consultation for a scalp thickening that was steadily evolving for nine years. Maceration and pruritus were the patient’s main concerns. Hairdressing became tricky and the patient also reported cosmetic concerns. No similar cases in the family were to be disclosed.

**Figure 1:**
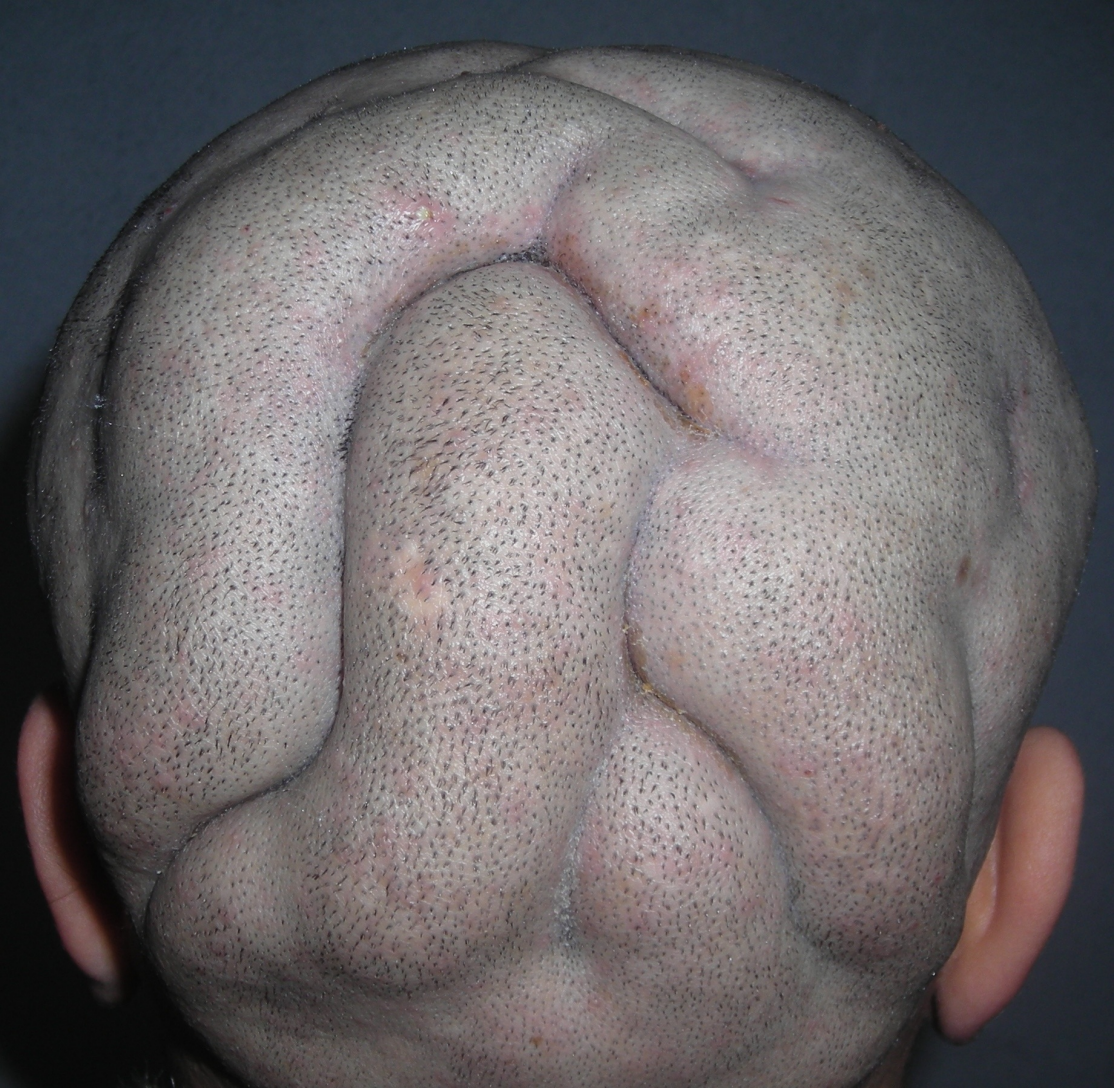
Longitudinal folds involving parietal and occipital areas.

The clinical examination found a soft and painless thickening of the scalp made of cerebriform convoluted folds and furrows, more conspicuous in the parietal and the occipital regions (Figure 1).

Otherwise, the patient appeared healthy. A punch scalp biopsy was then performed and did not reveal any inflammatory or neoplastic disease.

Blood screening tests including glycaemia, thyroid hormones, and growth hormone were all within normal ranges. Antinuclear antibodies and syphilis screening tests were negative.

Except for the scalp thickening, the computed tomography (CT)-scan of the head did not show any cranial or cerebral abnormality.

**Figure 2:**
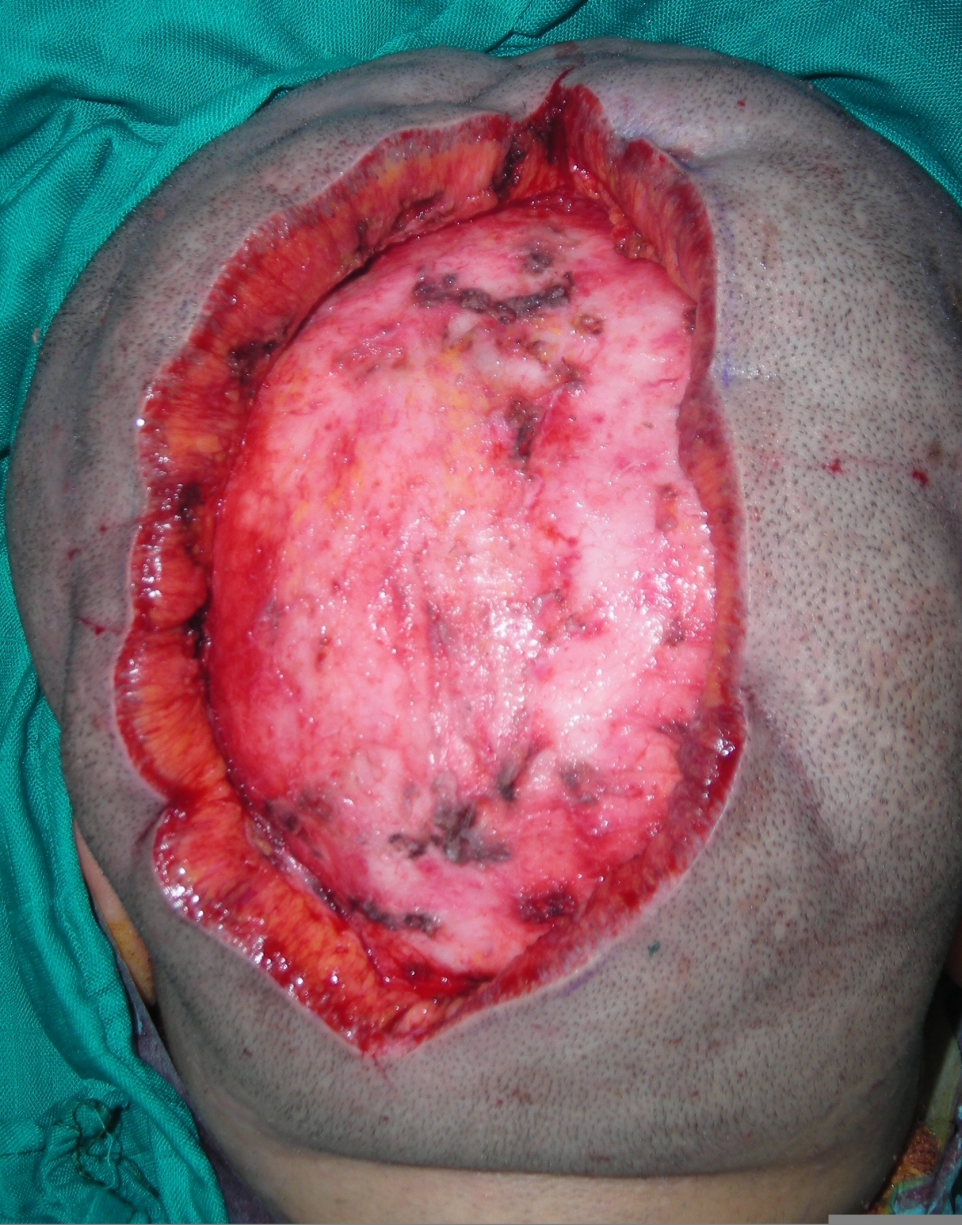
Longitudinal excision.

**Figure 3:**
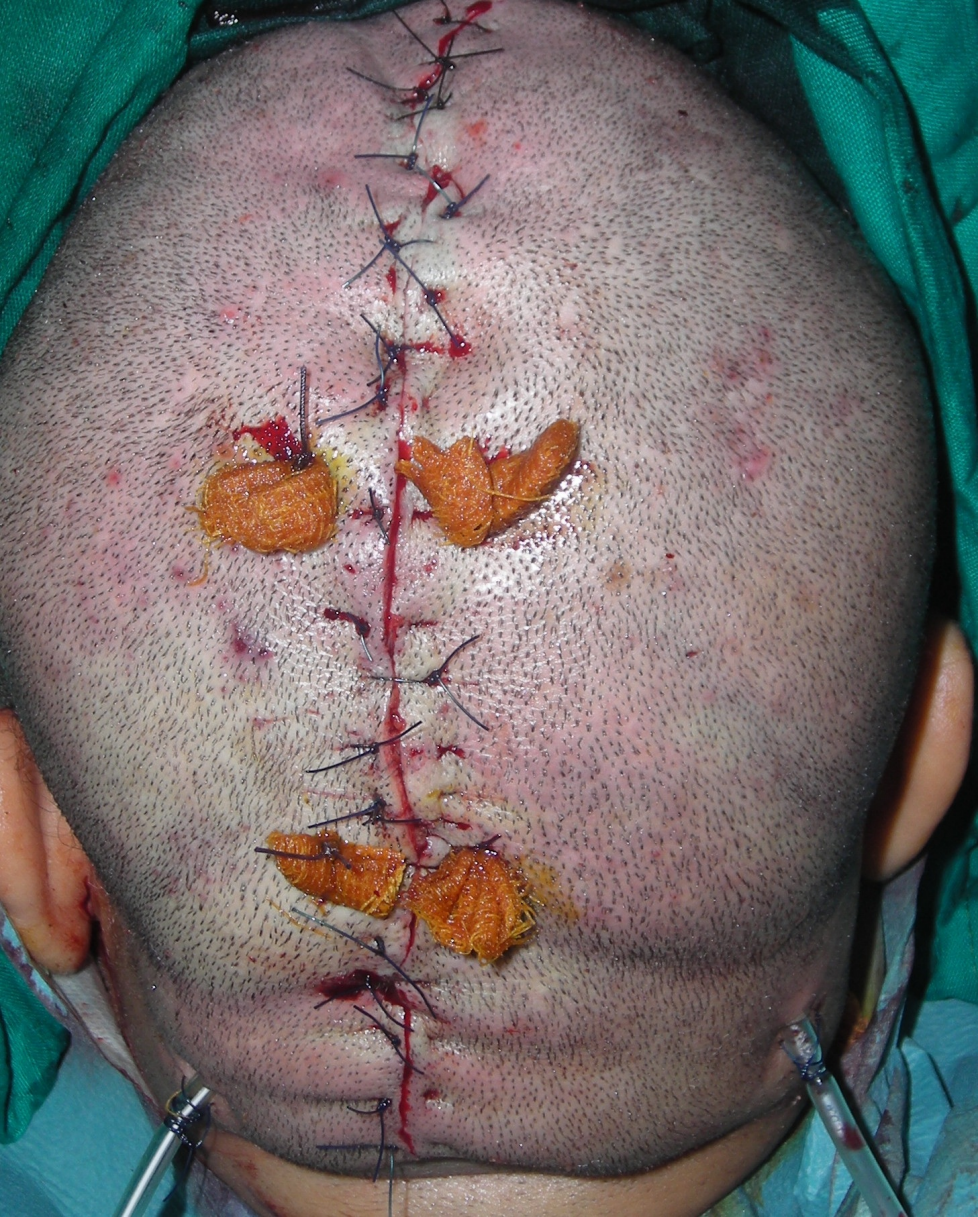
Direct closure.

In the light of these findings, the diagnosis of idiopathic CVG was raised. The patient was then admitted to the operating room for a partial surgical excision of the scalp under general anesthetic with oro-endotracheal intubation. She was laid down in a prone position to allow for easy access to the occipital extension of the scalp deformity. After the skin tension direction was checked and the tracing was made, about 50 mL of normal saline solution (0.9%) with epinephrine (0.4 mg) was infiltrated in the subgaleal and subcutaneous planes. An elliptical excision was carefully performed along the anteroposterior axis (Figure 2). The incision was made first on one lateral edge of the elliptical tracing. The dissection was carried out in the subgaleal plan and extended largely to the parietal and occipital regions. A basting stitch was put in the middle of the incision to ensure that the excision will allow for primary closure. The incision could be completed on the other edge allowing for the excision of a specimen 150 mm long and 60 mm wide, and direct closure (Figure 3) could then be achieved easily. The recovery and the follow-up were uneventful. One year later, the surgical outcome was satisfactory.

## Discussion

Since 1953, CVGs are classified into two main groups: secondary and primary [4].

Secondary CVG occurs as a consequence of conditions that produce pathological changes in the scalp structure [1]. Several local disorders (psoriasis, eczema, impetigo, tumors) or systemic disorders (acromegaly, diabetes mellitus, thyroid aplasia, syphilis, hyper IgE syndrome) have been reported to be in cause. It may occur at any age and both sexes are equally susceptible to this condition.

Primary CVG refers to cases with no underlying cause. Garden and Robinson [5] subclassified this entity into essential or idiopathic and non-essential. In the last one, associated neurological (epilepsy, intellectual disability, microcephaly), psychiatric (schizophrenia) or ophthalmic disorders (cataract, strabismus) are found, whereas in essential or idiopathic CVG no abnormalities are associated to the scalp “deformity”. The pathogenesis of this rare entity remains unclear. The male predominance and the postpubertal onset suggest an endocrine origin [2]. Although most cases are sporadic, some cases of familial primary CVG have been reported in the literature. Idiopathic CVG usually occurs before 30 years of age and affects predominantly men [3]. The scalp surface exhibits symmetrical folds, arranged in a sagittal direction. Most cases involve the vertex and the occiput only, however, the entire scalp may be affected.

The present paper reports the occurrence of an idiopathic CVG in a female patient which is extremely rare. To our knowledge, only one similar case [6] has been reported so far.

Asserting the diagnosis of CVG is based on clinical findings. However, complementary investigations such as skin biopsies, blood tests, and radiology examinations are recommended to distinguish between secondary and idiopathic forms of CVG. Histopathologic features of primary CVG range from normal skin structure to thickened connective tissue with hypertrophy or hyperplasia of adnexal structures [7].

Idiopathic CVG can be managed in different ways depending on the severity of the condition and the patients’ concerns. Abstention can be recommended for minor subclinical forms [3]. The medical approach based on the maintenance of a high level of local hygiene aims to prevent maceration and infections. The use of isotretinoin and corticoids in this indication did not show any conclusive results.

Surgery remains the best option for patients who experience complications such as maceration or infections and for those who express major psychological or cosmetic concerns. The surgical approach consists of a scalp reduction surgery. Some authors support the use of tissue expansion before the scalp reduction surgery [7] while others prefer excision and direct closure. Several excision patterns have been suggested. Dumas, et al. [8] reported a T-shaped excision pattern. Radwanski, et al. [9] favor a fleur-de-lis-shaped excision pattern. Anyway and whatever surgical technique is chosen, it must be as predictable and reliable as possible. The surgical technique must spare the scalp vascularization to prevent healing complications and to allow for a subsequent surgery if needed. In the present case, considering the anteroposterior direction of the grooves, we performed a longitudinal excision along the midline and parallel to the folds, leaving thus intact the main vascular pedicles of the scalp. This approach allowed for a good cosmetic outcome while preserving all possibilities for a subsequent surgical repair.

## Conclusions

In summary, because idiopathic CVG occurs exceptionally in women, it is recommended to rule out secondary forms. Good hygiene and surgical excision remain the adequate solution in complicated or advanced cases with important cosmetic and psychological prejudice.
